# Identification of Long Noncoding RNAs Associated With the Clinicopathological Features of Papillary Thyroid Carcinoma Complicated With Hashimoto’s Thyroiditis

**DOI:** 10.3389/fonc.2022.766016

**Published:** 2022-03-11

**Authors:** Yu Zhang, Kai-Ning Lu, Jin-Wang Ding, You Peng, Gang Pan, Li-Song Teng, Ding-Cun Luo

**Affiliations:** ^1^ Department of Oncological Surgery, Affiliated Hangzhou First People’s Hospital, Zhejiang University School of Medicine, Hangzhou, China; ^2^ Department of Head and Neck Surgery, Cancer Hospital of the University of Chinese Academy of Sciences, Hangzhou, China; ^3^ Cancer Center, The First Affiliated Hospital, Zhejiang University school of Medicine, Hangzhou, China

**Keywords:** Hashimoto’s thyroiditis, papillary thyroid carcinoma, clinicopathological features, long noncoding RNA, microarray

## Abstract

Long noncoding RNAs (lncRNAs) play a significant role in cancer biology. This study aimed to determine the roles of lncRNAs in establishing the differences in clinical features between patients with papillary thyroid carcinoma (PTC) without Hashimoto’s thyroiditis (HT) and patients with PTC and HT. In the present study, we detected the differentially expressed lncRNAs between tumor tissues of patients with PTC with or without HT through lncRNA microarrays. The data were verified and analyzed through qRT-PCR, cell viability, cell cycle and bioinformatics analyses. We found that 1031 lncRNAs and 1338 mRNAs were abnormally expressed in 5 tissue samples of PTC complicated with HT [PTC/HT (+)] compared with 5 samples of PTC without HT [PTC/HT (-)]. Gene Ontology and pathway analyses of the mRNAs suggested that several biological processes and pathways, particularly immune system processes, were induced in the PTC/HT (+) tissues. Twenty lncRNAs were verified in 31 PTC/HT (+) and 64 PTC/HT (-) specimens by qRT-PCR, and the results were consistent with the microarray data. Specifically, ENST00000452578, a downregulated lncRNA in PTC/HT(+), was negatively correlated with the tumor size. Cell viability assays revealed that ENST00000452578 could inhibit cell proliferation. Our results indicate that lncRNAs and mRNAs play an important role in establishing the different clinical characteristics between patients with PTC/HT(+) and patients with PTC/HT(-), and might provide new insights from the perspective of RNA for obtaining a further understanding of the clinical features related to PTC with HT.

## Introduction

Papillary thyroid cancer (PTC) is the most common subtype of thyroid cancer, which is the fourth most common cancer in Hispanic women in the USA, and over 586,000 cases worldwide (1, 2). Hashimoto’s thyroiditis (HT, also known as chronic lymphocytic thyroiditis, CLT) is the most common autoimmune thyroid disease, and its pathology is characterized by fibrosis, diffuse lymphocyte infiltration, and parenchymal atrophy. The disease occurs in 3–1.5 per 1000 individuals worldwide and the latest epidemiological survey in China shows the overall prevalence of positive thyroid antibodies was 14.19%, which is closely related to HT (3–5). Epidemiological data have indicated that HT frequently coexists with PTC (6). The association between HT and PTC was first described in 1955. Since then, an increasing number of studies have investigated the association between HT and PTC, and many studies showing that the clinicopathological features of PTC complicated with HT [PTC/HT (+)] are different from those of PTC without HT [PTC/HT (-)] (7–9). Compared with PTC/HT (-), PTC/HT (+) is associated with a lower VI lymph node metastasis rate, lower-stage disease, and less disease persistence, but the molecular mechanisms underlying these differences remain unelucidated.

Long noncoding RNAs (lncRNAs) are transcripts with a length of more than 200 nucleotides and extensive functions in biological processes and tumorigenesis (10–12). Some lncRNAs have been identified as important regulators of PTC. For instance, lncRNA LINC01614 affect PTC cell proliferation, migration, and invasion *in vitro*, this study also demonstrated LINC01614 as a novel oncogenic lncRNA in PTC (13). The lncRNA PTCSC3-mediated glycolysis suppresses thyroid cancer progression *via* interfering with PGK1 degradation and may be a potential therapeutic target for PTC treatment (14). However, the value of lncRNAs in the differences in clinicopathological features between PTC/HT (+) and PTC/HT(-) has not yet been investigated.

The aim of this study was to determine the roles of lncRNAs in the differences in clinical features between patients with PTC/HT (+) and patients with PTC/HT (-). An exploration of the related mechanisms may provide a more precise strategy for the diagnosis and treatment of PTC of different types and characteristics, particularly PTC with HT.

## Materials and Methods

### Patient Samples

A total of 105 thyroid tumors were obtained from surgical operations conducted at the Department of Surgical Oncology of the Affiliated Hangzhou First People’s Hospital, Zhejiang University School of Medicine from June 2016 to July 2018. Based on the clinicopathological features, the samples were further divided into two groups: PTC complicated with HT [PTC/HT (+)] and PTC without HT [PTC/HT (-)]. 10 samples [5 from PTC/HT (+) and 5 from PTC/HT (-)] were selected for lncRNAs microarray analysis, and the other 95 samples were used for further evaluation. None of the recruited donors had received chemotherapy, radiotherapy or other treatments before surgery. Participants with rheumatoid arthritis, chronic colitis, diabetes, renal disease, hepatic disease, mental disease, cardiovascular disease, other chronic diseases, or autoimmune diseases were excluded from this study. Most of the samples used in the study were fresh samples, and the central area of pure tumor tissue was cut immediately after surgical removal of the thyroid gland. A volume of approximately 0.3 cm*0.3 cm*0.2 cm can meet the experimental requirements (gray–white tissue that can be recognized by the naked eye is completely different from the surrounding normal thyroid tissue). The samples were then immediately placed in liquid nitrogen and placed in a refrigerator at -80°C until storage. From the very small number of samples with a very small tissue content, postoperative paraffin samples were used for RNA extraction. We performed tumor boundary and tumor scraping on the corresponding area of the white film according to the tumor tissue area in the HE-stained section.

All human tissue samples were obtained with patient consent and the study was approved by the Institutional Review Board of the Affiliated Hangzhou First People’s Hospital, Zhejiang University School of Medicine.

### Plasmids, Cell Culture, and Transfection

Plasmids were constructed through restriction digestion and ligation-mediated cloning and were verified by restriction digestion and sequencing prior to use. The human PTC cell lines TPC-1 and K1 were obtained from the American Type Culture Collection(ATCC) and cultured in RPMI 1640 medium (Invitrogen, Inc., Carlsbad, CA, USA) with 5% (v/v) fetal bovine serum (FBS) and 2 mM L-glutamine at 37°C in a humidified 5% CO_2_ incubator. The cell lines were authenticated based on the STR profile. Cell transfection was performed according to the manufacturer’s protocol (GeneChem).

### RNA Extraction

The tissue samples were placed in a mortar, and the tissues were rapidly abraded with liquid nitrogen. Total RNA was extracted from 105 tissue samples using RNAiso Plus (TaKaRa, Code: D9108A, Japan) according to the manufacturer’s protocol. The RNA purity was evaluated using a NanoDrop ND-2000 (Thermo, USA) spectrophotometer based on theOD260-to-OD280 ratio, and the RNA integrity was assessed by standard denaturing agarose gel electrophoresis.

### DNA Microarray

Arraystar Human lncRNA Microarray V3.0, which is designed for the global profiling of human lncRNAs and protein-coding transcripts was utilized in this study. Sample labeling and array hybridization were performed according to the Agilent One-Color Microarray-Based Gene Expression Analysis protocol (Agilent Technology) with minor modifications. Briefly, mRNA was purified from total RNA after the removal of rRNA (mRNA-ONLY™ Eukaryotic mRNA Isolation Kit, Epicenter). Each sample was then amplified and transcribed into fluorescent cRNA along the entire length of the transcripts without 3’ bias utilizing a random priming method. The labeled cRNAs were purified using an RNeasy Mini Kit (Qiagen). The concentration and specific activity of the labeled cRNAs (pmol Cy3/μg cRNA) were measured using a NanoDrop ND-1000 system. Subsequently, 1 μg of each labeled cRNA was fragmented by the addition of 5 μL of 10X Blocking Agent and 1 μL of 25X Fragmentation Buffer. The mixture was then heated to 60°C for 30 min. Finally, 25 μL of 2X GE Hybridization buffer was then added to dilute the labeled cRNA. Subsequently, 50 μL of hybridization solution was dispensed into the gasket slide, and this slide was then placed on the lncRNA expression microarray slide. The slides were incubated for 17 h at 65°C in an Agilent hybridization oven, and the hybridized arrays were washed, fixed and scanned using an Agilent DNA Microarray Scanner (part number G2505C).

### Microarray Data Analysis

Agilent Feature Extraction software (version 11.0.1.1) was used to analyze the acquired array images. Quantile normalization and subsequent data processing were performed using the GeneSpring GX v11.5 software package (Agilent Technologies). After quantile normalization of the raw data, lncRNAs and mRNAs from at least 5 of 10 samples with flags in Present or Marginal (“All Targets Value”) were selected for further data analysis. The lncRNAs and mRNAs showing a significant difference between the two groups were identified by fold-change filtering and volcano plot filtering. Hierarchical clustering was performed using Agilent GeneSpring GX software (version 11.5). We used t tests, and the fold change cutoff was 4.0. The association between the expression levels of each lncRNA was evaluated by the Benjamini–Hochberg FDR multiple testing correction. lncRNAs were considered differentially expressed if their adjusted p values were less than 0.05. GO analysis is a functional analysis that associates differentially expressed mRNAs with GO categories, and these GO categories are derived from Gene Ontology (www.geneontology.org), which comprises three structured networks of defined terms that describe gene product attributes. GO and pathway analyses were performed using the standard enrichment computation method.

### Construction of the LncRNA-mRNA Coexpression Network

A coding-noncoding gene coexpression (CNC) network was constructed based on a correlation analysis between the differentially expressed lncRNAs and mRNA molecules. For each pair of genes, a Pearson correlation coefficient was calculated, and significant correlation pairs were selected to construct the network. The network construction procedures included calculation of the Pearson correlation coefficients and use of R values to determine the correlation coefficient of the Pearson correlation coefficients (PCCs) between the lncRNA coding genes. lncRNAs and mRNAs with PCC≥0.95 were selected to construct the CNC network using the open source bioinformatics software program Cytoscape (v2.8.1). Based on the latest Kyoto Encyclopedia of Genes and Genomes(KEGG, http://www.genome.jp/kegg) database, we performed a pathway analysis of the differentially expressed mRNAs. This analysis enabled the identification of biological pathways with significant enrichment of the differentially expressed mRNAs (the P value cutoff was 0.05). We estimated the potential functions of lncRNAs according to the results from the KEGG and GO analyses or their related mRNAs.

### Quantitative Real-Time Polymerase Chain Reaction (qRT–PCR) Validation

Total RNA was reverse-transcribed using the PrimeScript™ RT reagent kit with gDNA Eraser (TaKaRa, RR047A, Japan) according to the manufacturer’s protocol. qRT–PCR was performed with SYBR^®^ Premix Ex TaqTM II (TaKaRa, RR420A, Japan) and an ABI 7500 PCR (USA) instrument according to the manufacturer’s instructions. The composition of the 25-μL reaction was as follows: 12.5 μL of SYBR^®^ Premix Ex Taq™ II (Tli RNase Plus) (2×), 0.4 μL of the PCR forward primer (10 μM), 0.4 μL of the PCR reverse primer (10 μM), 0.2 μL of ROX reference dye II (50×), 3 μL of the cDNA solution, and 8.5 μL of double-distilled water. The reaction solution was placed on ice. All experiments were performed in triplicate, and each set of samples included a blank control. Twenty lncRNAs that were significantly deregulated were evaluated, and GAPDH was used as an internal control. The primers of the genes tested are listed in **Tables S1**, **S2**. For visualization of the quantitative results, the expression of each lncRNA was represented as the fold-change calculated using the 2^-ΔCT^ method (15).

### MTT Assay

Cells were seeded in a 96-well plate and incubated for 24h. After treatment, the cells were incubated with 50 μl of 1 mg/mL 3-(4,5-dimethylthiazol-2-yl)-2,5-diphenyltetrazolium bromide (MTT, Sigma–Aldrich Chemical) in PBS for 3 h according to the manufacturer’s instructions. Purple formazan was then solubilized in DMSO, and the absorbance at 570 nm was read using a microplate reader.

### Cell Cycle Analysis

After the treatment, the cells were fixed with ice-cold 70% ethanol and stored at 4°C (or -20°C) for at least 4 h. The cells were washed with PBS, treated with 1 mg/mL RNase A and incubated at 37°C for 30 min. The cells were then stained with 1 mg/mL PI, incubated at RT for 30 min in the dark and analyzed by flow cytometry.

### Statistical Analysis

All statistical analyses were performed using the software package SPSS 19.0 (SPSS Inc., Chicago, IL, USA). The Kolmogorov–Smirnov (K-S) test was used to determine whether the data obtained were normally distributed. Normally distributed data are presented as the mean ± standard deviations (SDs), and were analyzed by one-way analysis of variance (ANOVA) or independent-sample t tests. If the assumption of equal variance was not met, the nonparametric Mann–Whitney U test was used for the comparison. P<0.05 was considered to indicate statistical significance.

## Results

### Clinicopathological Characteristics

The 105 patients included 11 male and 94 female patients and had a median age of 43 years (range, 8–74 years); in addition, 69 of the patients were classified as PTC/HT (-), and the other 36 were PTC/HT (+). The mean ages of PTC/HT (+) and PTC/HT (-) were 42.97 ± 12.48 and 44.01 ± 13.32 years, respectively. The mean tumor sizes of PTC/HT(+) and PTC/HT(-) patients were 1.27 ± 0.78 cm and 0.89 ± 0.46 cm, respectively (P<0.05); The mean thyroid stimulating hormone (TSH) level (mU/L) in PTC/HT (+) and PTC/HT (-) was 3.41 ± 2.72 and 2.22 ± 1.27 (P<0.05), and the mean numbers of foci in these patients were 1.78 ± 0.93 and 1.26 ± 0.61 respectively (P<0.01). For patients with multiple lesions, we routinely obtained the lesion with the largest diameter for genetic testing and comparison between samples. The demographic and clinical characteristics of the patients included in this study are shown in **Table 1**.

**Table 1:** The demographic and clinical characteristics of the enrolled patients.

Clinical parameter	PTC/HT(+) (n = 36 per group)	PTC/HT(-) (n = 69 per group)	P value
Age (years)	42.97 ± 12.48	45.01 ± 13.32	0.329
Gender			
*Female*	33	61	0.606
*Male*	3	8	
TSH(mU/L)	3.41 ± 3.72	2.22 ± 1.27	0.041*
Number of foci	1.78 ± 0.93	1.26 ± 0.61	0.002**
Tumor size(cm)	1.27 ± 0.78	0.89 ± 0.46	0.022*
Extrathyroidal extension			
*No*	30	49	0.167
*Yes*	6	20	
CLNM			
*No*	20	40	0.813
*Yes*	16	29	
Microcarcinoma			
*No*	18	31	0.623
*Yes*	18	38	
TNM stage			
*I-II*	31	53	0.260
*III-IV*	5	16	

TSH, thyroid stimulating hormone; * indicates P < 0.05, ** indicates P < 0.01. CLNM, central compartment lymph node metastasis; TNM stage, tumor-node-metastasis stage.

### Overview of the LncRNA and Subgroup Analyses

**Figure 1:**
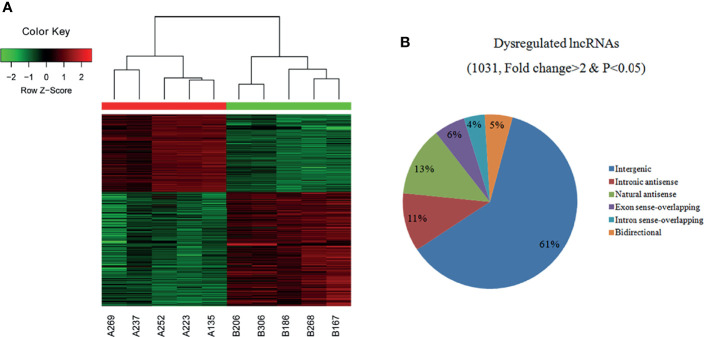
Heat maps showing the differential expression and hierarchical clustering of lncRNAs **(A)** in PTC/HT (+) versus PTC/HT (-). “Red” indicates high relative expression, and “green” indicates low relative expression. The classification and proportions of dysregulated lncRNAs are shown according to their relationships with their associated protein-coding genes **(B)**.

A total of 30,586 lncRNAs were detected in the PTC/HT (+) and PTC/HT (-) tissues, but only thousands of lncRNAs were significantly upregulated or downregulated. A cluster analysis arranges samples into groups based on their expression levels to facilitate the formulation of hypotheses regarding the relationships among samples. The dendrogram in **Figure 1A** shows the lncRNA expression patterns in the PTC/HT(+) and PTC/HT(-) tissues. The samples were distinctly separated into two groups [e.g., PTC/HT(+) and PTC/HT(-)] according to the lncRNA expression patterns. Based on the lncRNA expression profiles, 1031 differentially expressed lncRNA transcripts were identified from the comparison of the PTC/HT (+) and PTC/HT(-) tissues (≥2-fold), and these included 415 and 616 lncRNAs that were significantly (≥2-fold) upregulated and significantly downregulated in the PTC/HT(+) tissues compared with the PTC/HT(-) tissues. The expression of uc021set.1 (fold change A/B=108.21) exhibited the most significant upregulation, whereas the expression of ENST00000539568 (fold change B/A=12.36) showed the most significantly downregulation.

The dysregulated lncRNAs were classified into six subgroups (intergenic, intronic antisense, natural antisense, exon sense-overlapping, bidirectional and intron sense-overlapping) according to their relationships with their associated protein-coding genes as shown in **Figure 1B**.

### Overview of mRNA Profiles

**Figure 2:**
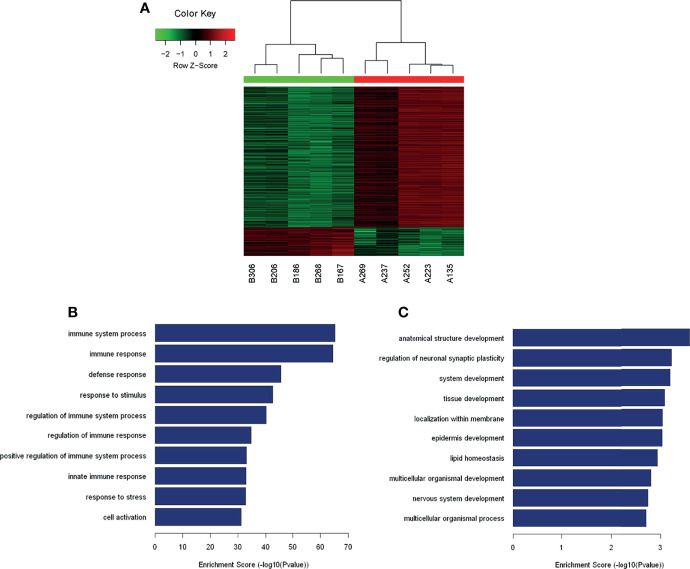
Heat maps showing the differential expression and hierarchical clustering of mRNAs **(A)**. The top10 up-regulated and down-regulated pathways are presented **(B, C)**.

The analysis of the identified transcripts with the differential (≥2-fold) expression showed that 1154 upregulated and 234 downregulated mRNAs exhibited a significant difference between the PTC/HT (+) and PTC/HT (-) tissues. The expression of IGJ (fold change A/B=50.44) exhibited the strongest upregulation, whereas the expression of NASP (fold change B/A=13.43) showed the highest downregulated (**Table 2**). A clustering analysis revealed the relationships among the mRNA expression patterns between PTC/HT(+) and PTC/HT(-) (**Figure 2A**).

**Table 2:** Deregulated mRNAs detected in PTC/HT(+) and PTC/HT(-).

Up-regulated		Down-regulated	
mRNAs	Fold change (A/B)	mRNAs	Fold change (B/A)
IGJ	50.4404	NASP	13.4315
MZB1	41.5173	CYP4B1	8.4285
CXCL13	39.8112	NMU	6.5480
SLC6A14	27.4231	SYT12	6.4047
COL11A1	27.3843	CD22	6.4047
SLAMF7	23.9819	ZNF534	6.2924
GZMK	23.9540	ZCCHC16	6.1796
FCRL5	22.0986	C1orf115	6.0817
GZMH	16.2954	SOWAHA	6.0744
CXCL10	15.3891	KLK7	5.3778

### GO and Pathway Analyses

A GO analysis constitutes a functional analysis of differentially expressed mRNAs associated with different GO categories. The GO categories are derived from Gene Ontology (www.geneontology.org), and a GO analysis was performed to determine the enrichment of genes and gene products involved in biological processes, cellular components and molecular functions. The GO analysis indicated that the transcripts showing significant differential expression between PTC/HT(+) and PTC/HT(-) were predominantly associated with immune system processes (ontology: biological processes), protein binding (ontology: molecular functions), and the plasma membrane (ontology: cellular components). The GO analysis of dysregulated mRNAs in PTC/HT(+) revealed the involvement of biological processes and signaling pathways (**Figure S1**). Interestingly, most of the upregulated mRNAs were associated with the immune response. The top 10 upregulated and downregulated biological processes are shown in **Figures 2B, C**, respectively.

The pathway analysis identified 7 downregulated pathways (**Table S3**), and the most enriched network was ‘Circadian rhythm-Homo sapiens (human)’, which is composed of 4 targeted genes. Furthermore, the analysis revealed that of the 57 upregulated pathways (**Table S4**) identified, the most enriched network was ‘Staphylococcus aureus infection-Homo sapiens (human)’, which was composed of 27 targeted genes.

### qRT–PCR Validation

**Figure 3:**
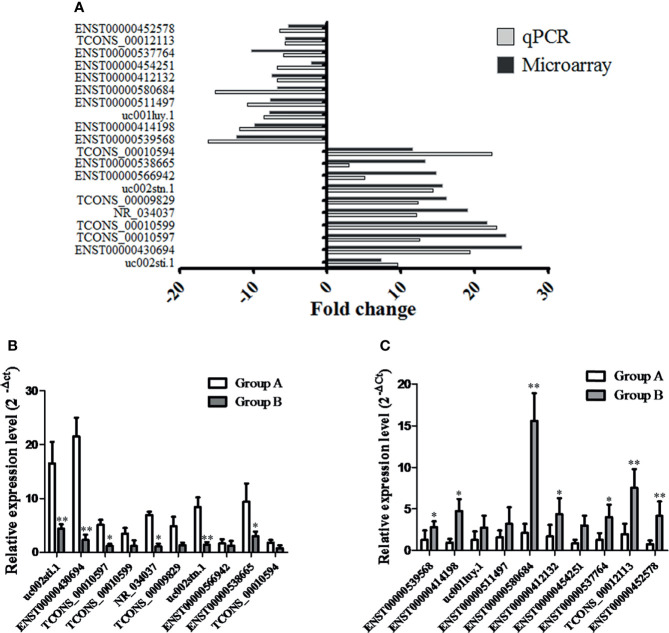
The differential expression of 20 lncRNAs observed by microarray was validated by qRT-PCR. Fold changes were calculated using the 2-ΔCt method. The heights of the columns in the chart represent the median fold changes in expression of each of the validated lncRNAs across the patients **(A)**. The relative expression of up-regulated lncRNAs and down-regulated lncRNAs are presented in **(B, C)**, respectively. Data are presented as the mean ± SD. GAPDH was used as a control. * indicates P < 0.05. ** indicates P < 0.01. Group A indicates PTC/HT (+), Group B indicates PTC/HT (-).

Based on the fold-change data (16), we subsequently validated the changes in top 20 lncRNAs, including 10 lncRNAs with an upregulated fold-change >10 (as listed in **Table S1**) and 10 lncRNAs with an downregulated fold-change >5 (as listed in **Table S2**); to that end, we determined their expression levels in 95 tissue samples (PTC/HT(+): 31; PTC/HT(-): 64) by qRT–PCR. The qRT–PCR results revealed different degrees of agreement between the qRT–PCR results and the lncRNA expression levels identified from the microarray data (**Figure 3A**). The relative expression levels of lncRNAs are presented in **Figure 3B** (upregulated lncRNAs) and **Figure 3C** (downregulated lncRNAs). 6 lncRNAs (uc002sti.1, ENST00000430694, TCONS_00010597, NR_034037, uc002stn.1, ENST00000538665) were upregulated and 7 lncRNAs (ENST00000539568, ENST00000414198, ENST00000580684, ENST00000412132, ENST00000537764, TCONS_00012113, ENST00000452578) were downregulated in the PTC/HT(+) tissue samples compared with the PTC/HT(-) tissue samples, and these findings were in good agreement with the microarray results.

### Functional Analysis of the LncRNA ENST00000452578

To further select more credible lncRNAs for the further analysis, 13 lncRNAs were filtered using a database of RNA-seq-based lincRNA expression levels in thyroid tissue included in the UCSC (https://genome.ucsc.edu/). According to the results, five lncRNAs (ENST00000539568, ENST00000414198, ENST00000580684, TCONS_00012113 and ENST00000452578) were expressed at high levels in thyroid tissue, and the association analysis between the expression of the above five lncRNAs and the clinical characteristics of tumors found that only ENST00000452578 was negatively correlated with tumor size (Mann-Whitney U-test, Z = 2.659, P = 0.008; **Table 3**), which indicates that ENST00000452578 may play a negative regulatory role in tumor cell proliferation and was chosen for further analysis.

**Table 3:** Relationship between expression level of lncRNA ENST00000452578 and clinical features.

Clinical figures	Group	n	ENST00000452578 Expression Level	P value
Age (years)	≥45	46	2.04 ± 2.57	0.127
	<45	49	1.40 ± 1.88	
Gender	Female	85	1.81 ± 2.34	0.139
	Male	10	0.86 ± 0.10	
Number of lesions	Single lesion	73	1.59 ± 2.10	0.600
	Multiple lesions	22	2.12 ± 2.71	
Tumor size(cm)	≤1	57	2.26 ± 2.72	0.008^**^
	>1	38	0.89 ± 0.74	
Extrathyroidal extension	No	69	1.73 ± 2.33	0.465
	Yes	26	1.68 ± 2.08	
CLNM	No	53	1.69 ± 2.34	0.658
	Yes	42	1.74 ± 2.17	
TNM stage	I-II	73	1.98 ± 2.02	0.539
	III-IV	22	2.05 ± 2.58	

The expression values were calculated by (2^-ΔCT^). lncRNA expression levels are presented as the mean ± SD. ^**^ indicates P < 0.01. AJCC TNM staging, seventh edition.

**Figure 4:**
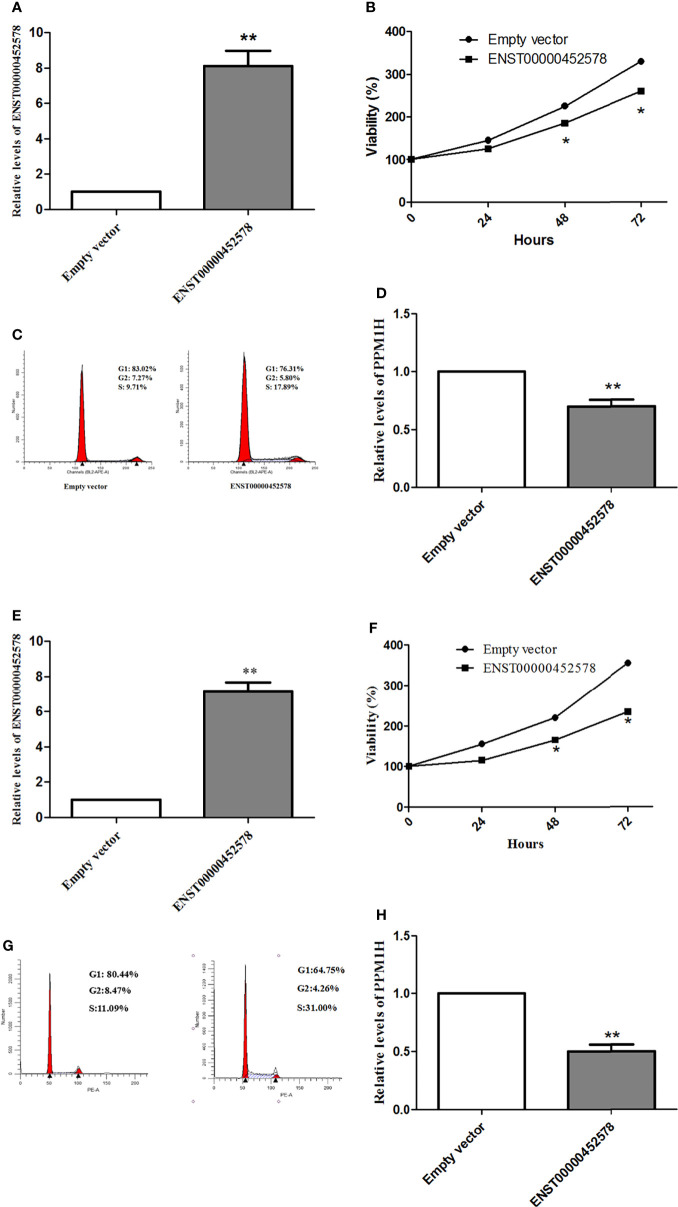
Functional analysis of the lncRNA ENST00000452578 in the PTC cell line TPC-1 **(A–D)** and K1 **(E–H)**. **(A, E)**, cells were transfected with the ENST00000452578 overexpression plasmid and validated by qRT-PCR; **(B, F)**, MTT assays were performed to detect the cell growth at the indicated time points after transfection; **(C, G)**, cell cycle analysis was performed in 48h after transfection; **(D, H)**, qRT-PCR was used to detect the PPH1M expression in 48h after transfection. Data are presented as the mean ± SD, n=3. *P < 0.05 vs. control, **P < 0.01 vs. control.

Subsequently, we tested whether the dysregulation of the lncRNA ENST00000452578 contributes to the development of PTC. An ENST00000452578 overexpression plasmid was constructed and transfected into the human PTC cell lines TPC-1 and K1. ENST00000452578 expression was significantly higher in these transfected than in TPC-1 (t test, t=8.601, P=0.0013; **Figure 4A**) and K1 (t test, t=6.126, P=0.0058; **Figure 4E**) cells with the empty vector control. As predicted, the high level of ENST00000452578 effectively suppressed the growth of TPC-1 cells (t test, t=6.385, P=0.0237 at 72 h; **Figure 4B**) and K1 cells (t test, t=8.586, P=0.0168 for 72 h; **Figure 4F**), as demonstrated by cell cycle arrest at the S phase (**Figures 4C, G**). To ascertain the correlation between the differentially expressed lncRNAs and mRNAs in PTC/HT(+) and PTC/HT(-), we constructed a lncRNA-mRNA coexpression network. In addition, a coding-noncoding gene coexpression network was generated based on the correlation analysis between the differentially expressed lncRNAs and mRNAs. Some lncRNAs showing obvious differential expression were selected to perform a coexpression network analysis using Cytoscape. Among the CNC networks, the lncRNA ENST00000452578 was correlated with 9 mRNAs, namely, PITPNC1, METTL7B, PPM1H, SYT12, CAMP, TIPARP, OLFM1, ICAM5, and SNAP25. Among these mRNAs, the expression of protein phosphatase 1H (PPM1H) in PTC/HT(+) is higher than PTC/HT(-), which is negatively correlated with the expression of ENST00000452578. PPM1H has been implicated in various intracellular processes that are relevant to cancer, including cell cycle regulation (17, 18). In our study, PPM1H was significantly downregulated following ENST00000452578 overexpression in BHP 2-7 cells (t test, t=5.196, P=0.0035; **Figure 4D**) and K1 cells (t test, t=9.296, P=0.0019; **Figure 4H**). These findings indicate that PPM1H may be negatively regulated by ENST00000452578.

## Discussion

Our study implies that the clinicopathological features of PTC/HT(+) are different from those of PTC/HT(-), which is similar to other results (7–9, 19, 20) In addition, our result indicates that lncRNAs and mRNAs may play an important role in establishing the different clinical characteristics between patients with PTC/HT(+) and PTC/HT(-). If we can find out the specific mechanism, it may bring new clinical methods to the diagnosis and treatment of PTC combined HT.

According to recent studies, the possible mechanisms is partly due to the modulation of the tumor microenvironment, the induction of immune responses and the presence and low frequency of BRAF mutations (21–24). At the same time, the GO analysis in our study indicated that the transcripts showing significant differential expression between PTC/HT(+) and PTC/HT(-) were predominantly associated with immune system processes, such as T cell proliferation, regulation of lymphocyte activation and so on. However, our study did not explain how the T cell proliferation and other immune system participate in this process. More recent studies by a retrospective cohort study suggest that the imbalance between cytotoxic and regulatory T lymphocytes(CD8+/Foxp3 T lymphocyte ratio) in the peri-tumoral PTC/HT(-) may affect the tumor-specific immune response. Moreover, the number of Tregs while an inverse correlation with CD8+/Foxp3+ T cells ratio was observed in PTC/HT(+) (25). These data suggest that regulatory T lymphocytes in the peri-tumor may afect behavior of cancer. However, above studies are all retrospective studies, then a prospective​studies which generated a mouse model where PTC and thyroiditis develop in a predictable manner, combining the oncogenic drive of the BRAF-v600E mutation to the thyroiditis susceptibility of the NOD.H2h4 strain, suggests that the intratumoral mononuclear cell infiltration was more prominent in mice with pre-existing thyroiditis and sustained by a significant expansion of effector memory CD8 + T cells and CD19 + B cells (26). These further suggests that the immune response may play an important role in the different clinical characteristics between patients with PTC/HT(+) and PTC/HT(-), possibly through the intratumoral mononuclear cell infiltration, then sustained by a significant expansion of regulatory T lymphocytes. Afterwards, the reason for the better prognosis of PTC patients with concurrent HT maybe due to B lymphocyte–related immune response (27). Unfortunately, the sample size in this study is too small (only 3 cases of PTC/HT(+)), which cannot fully explain the problem, more samples are needed in the future. However, all these findings shed light on the possible PTC-thyroiditis association and emphasize the contribution of intratumoral T and B lymphocytes to the difference between patients with PTC/HT(+) and PTC/HT(-).

In addition, many lncRNAs also play a significant role in the regulation of immunity, such as lncRNA UCA1 attenuated the killing effect of cytotoxic CD8 + T cells on anaplastic thyroid carcinoma (ATC) cells through the miR-148a/PD-L1 pathway (28). Afterwards, further studies confirmed that lncRNA NONHSAT079547.2 could promote cell growth and control IL-17 expression and secretion *via* the NONHSAT079547.2/miR-4716-5p/IL-17 axis in CD4+ T cells of HT patients (29). However, these lncRNAs were not found in our lncRNA microarrays, which may be due to different regulatory mechanisms bewteen PTC/HT(+) and PTC/HT(-). Furthermore, our screening results indicate that the novel lncRNA ENST00000452578 was significantly and negatively associated with the tumor size. Other studies have not seen the relationship between ENST00000452578 and tumors, but they have found that many other lncRNAs are related to tumor size in PTC, such as the novel lncRNA n384546 promotes PTC progression and metastasis by acting as a competing endogenous RNA of miR-145-5p to regulate AKT3 (30). Unfortunately, we still not found dysregulated expression of lncRNA n384546 in our results. After exploring the mechanism by which ENST00000452578 regulates tumor size, we found that ENST00000452578 may regulate the tumor size by regulating PPM1H. Hua Xu et al (31) implied that progression of glioma could restrain through targeting PPM1H through sponge of miR-424-5p. A similar mechanism may also exist in our study, further investigations should be performed to elucidate the function of ENST00000452578 and its underlying mechanisms.

What’s more, base on the results of microarray-based profiling performed in the present study identified many lncRNAs and mRNAs in PTC/HT(+) compared with PTC/HT(-), the pathway analyses identified some dysregulated pathways. Among them, we noticed the PI3K-Akt signaling pathway, which play a importent role in cell proliferation and migration in PTC (32–34). Many lncRNAs also play a significant regulatory role in PI3K-Akt signaling pathway in PTC (35), such as lncRNA XIST (36), lncRNA ABHD11-AS1 (37), lncRNA TTN-AS1 (38), etc. However, these lncRNAs were not found in our results for the same reason. What’s more, there are few reports on the PI3K-Akt signaling pathway in HT. The literature suggests that Prunella vulgaris may play an anti-inflammatory role by regulating the PI3K-Akt signaling pathway, thus realizing the treatment of HT (39). but this study only draws the conclusion through bioinformatics analysis, and has no *in vivo* or *in vitro* evidence. However, the PI3K-Akt signaling pathway was one of the most enriched networks. We speculated that the differences in clinical features between PTC/HT(+) and PTC/HT(-) might be associated with the PI3K-Akt signaling pathway, and the specific mechanism needs to be further tested and confirmed.

This study has limitations. First, in the study, the sample size was to small to further elucidate the role and mechanism of lncRNAs in the two groups. Second, we have no follow-up data of patients, so the relationship with prognosis is unclear and we will supplement this information in future research. Third, we did not obtain PTC cells in the background of HT, which made it difficult to distinguish the real reasons. Howerer, these problems could be solved if we expand the sample size and identify an approach to obtain pure tumor cells for further exploration.

In conclusion, our results provide the first indication that lncRNAs and mRNAs are dysregulated in PTC/HT(+) and PTC/HT(-) based on a microarray analysis, and these results might provide new insights for obtaining a better understanding of the differences in clinicopathological features between PTC/HT(+) and PTC/HT(-). Among them, ENST00000452578 was found to be closely related to tumor diameter between PTC/HT (+) and PTC/HT (-). However, additional investigations are required to confirm and expand these findings.

## Data Availability Statement

The original contributions presented in the study are publicly available. This data can be found here: NCBI GEO, GSE192560.

## Ethics Statement

The studies involving human participants were reviewed and approved by Ethics Committee of Hangzhou First People’s Hospital. The patients/participants provided their written informed consent to participate in this study.

## Author Contributions

YZ, conception and design, provision of study materials or patients, data analysis and interpretation, manuscript writing, and final approval of manuscript. K-NL and J-WD, conception and design, provision of study materials or patients, data analysis and interpretation, and final approval of manuscript. YP and GP, conception and design, provision of study materials or patients, and final approval of manuscript. L-ST, conception and design, manuscript writing and final approval of the manuscript. D-CL, conception and design, administrative support, provision of study materials or patients, manuscript writing, and final approval of manuscript. All authors contributed to the article and approved the submitted version. Here we would also like to thank Jianguo Sun for his contribution to the ideas of this paper.

## Funding

This work was supported by the Key Project of Medical Scientific and Technology Program in Hangzhou (grant number 2013Z04), the Project of Medical Scientific and Technology Program in Hangzhou (grant number A20200432), the Medical and Health Research Program of Zhejiang Province (2019RC240) and the Key Project of Scientific and Technology of Hangzhou Health Commission (OO20190490).

## Conflict of Interest

The authors declare that the research was conducted in the absence of any commercial or financial relationships that could be construed as a potential conflict of interest.

## Publisher’s Note

All claims expressed in this article are solely those of the authors and do not necessarily represent those of their affiliated organizations, or those of the publisher, the editors and the reviewers. Any product that may be evaluated in this article, or claim that may be made by its manufacturer, is not guaranteed or endorsed by the publisher.
