# Supplementary material for: Identification of Long Noncoding RNAs Associated With the Clinicopathological Features of Papillary Thyroid Carcinoma Complicated With Hashimoto’s Thyroiditis
Source: Front Oncol. 2022 Mar 11;12:766016. doi: 10.3389/fonc.2022.766016 (PMC8963332; doi:10.3389/fonc.2022.766016)
Supplement: Supplementary file 1 [file DataSheet_1.zip › revised-Supplementary Materials/revised-supplementary table S1.docx]

| **Up-regulated lncRNA** | **PCR Forward Primer** | **PCR Reverse Primer** |
| --- | --- | --- |
| **uc002sti.1** | TACTGAGCAAAACAACAC | AAACTCTCCCTAAGACTG |
| **ENST00000430694** | GAGTGTTAGAGGGAGAAGTG | AAAGCCAAGGAGGAGGAG |
| **TCONS_00010597** | GGTGAGTTACTTTGTGAACG | CACACTAACCCACCGACT |
| **TCONS_00010599** | GAGTTACTTTGTGAACGAG | ACACACTAACCCACCGAC |
| **NR_034037** | ATGCTCTGAAGGAAGGGA | TAAGAAATTCTGGAATGGG |
| **TCONS_00009829** | ATGAGTGGCTGTGAACGA | TCACATACTAACTCGCCC |
| **uc002stn.1** | TCCACTCTCTCTGTCCGTC | AATAAACCCCAACATCCTC |
| **ENST00000566942** | GTTAATGTCCTTTTGGTC | AATAGGTATAGTCGCCTC |
| **ENST00000538665** | CTGCAATTTCAGGTAGCTTTTC | TCAGAAAAGCTACCTGAAATTG |
| **TCONS_00010594** | GGGTGAGTTACTGTGTGAA | GCACTAACCCACTCTTTC |

Table S1. Primer sequences for PCR of 10 up-regulated lncRNAs

**Article title:** Identification of long noncoding RNAs associated with the clinicopathological features of papillary thyroid carcinoma complicated with Hashimoto's thyroiditis

**Author names:** Yu Zhang, Kai-ning Lu , Jin-wang Ding , You Peng, Gang Pan, Ding-cun Luo

**Affiliation and e-mail address of the corresponding author:**

Correspondence should be addressed to Dingcun Luo; ldc65@163.com

*Department of Oncological Surgery,* *Affiliated Hangzhou First People’s Hospital, Zhejiang University School of Medicine, Hangzhou, Zhejiang, 310006, China*
