# Supplementary material for: Identification of Long Noncoding RNAs Associated With the Clinicopathological Features of Papillary Thyroid Carcinoma Complicated With Hashimoto’s Thyroiditis
Source: Front Oncol. 2022 Mar 11;12:766016. doi: 10.3389/fonc.2022.766016 (PMC8963332; doi:10.3389/fonc.2022.766016)
Supplement: Supplementary file 1 [file DataSheet_1.zip › revised-Supplementary Materials/revised-supplementary table S2.docx]

| **Down-regulated lncRNA** | **PCR Forward Primer** | **PCR Reverse Primer** |
| --- | --- | --- |
| **ENST00000539568** | **AAGAAGAAGATGAAGTAGGGGA** | TGGAAAGTGTCAGGAGGGA |
| **ENST00000414198** | CCAGGCAAAATGGCAGTTC | GCTTCCTGTCAAGATGGAGAAA |
| **uc001luy.1** | TCTG**GG**CTTGGTCCTCTTG | CCTCCACCCTCCTCCTCC |
| **ENST00000511497** | TCATTGTTACAGCCCATC | AAGTTTACTGACACATCTTCTC |
| **ENST00000580684** | CGGTGTCTGGGGAGCGGCTGCCG | CCGGCAGCCGCTCCCCAGACACC |
| **ENST00000412132** | CTCTCTTCAACCAAGTAAAGGAGA | GTCTCCTTTACTTGGTTGAAGAGA |
| **ENST00000454251** | ATGAGGAGCAAAAACACA | CCTAAAAGGAATACGGAGT |
| **ENST00000537764** | CAAGGGCAGAATGAAGTC | AGATGATGAAAAATATTAAGAT |
| **TCONS_00012113** | AGGAGCCTGAAGAGAAAA | GCCAAGCATAGTGATAGC |
| **ENST00000452578** | GGACGTTCACCACCCAGAAC | CCGGGTCACAGGCTTGTATT |

Table S2. Primer sequences for PCR of 10 down-regulated lncRNAs

**Article title:** Identification of long noncoding RNAs associated with the clinicopathological features of papillary thyroid carcinoma complicated with Hashimoto's thyroiditis

**Author names:** Yu Zhang, Kai-ning Lu , Jin-wang Ding , You Peng, Gang Pan, Ding-cun Luo

**Affiliation and e-mail address of the corresponding author:**

Correspondence should be addressed to Dingcun Luo; ldc65@163.com

*Department of Oncological Surgery,* *Affiliated Hangzhou First People’s Hospital, Zhejiang University School of Medicine, Hangzhou, Zhejiang, 310006, China*
