# Supplementary figures and images for: Identification of Long Noncoding RNAs Associated With the Clinicopathological Features of Papillary Thyroid Carcinoma Complicated With Hashimoto’s Thyroiditis
Source: Front Oncol. 2022 Mar 11;12:766016. doi: 10.3389/fonc.2022.766016 (PMC8963332; doi:10.3389/fonc.2022.766016)

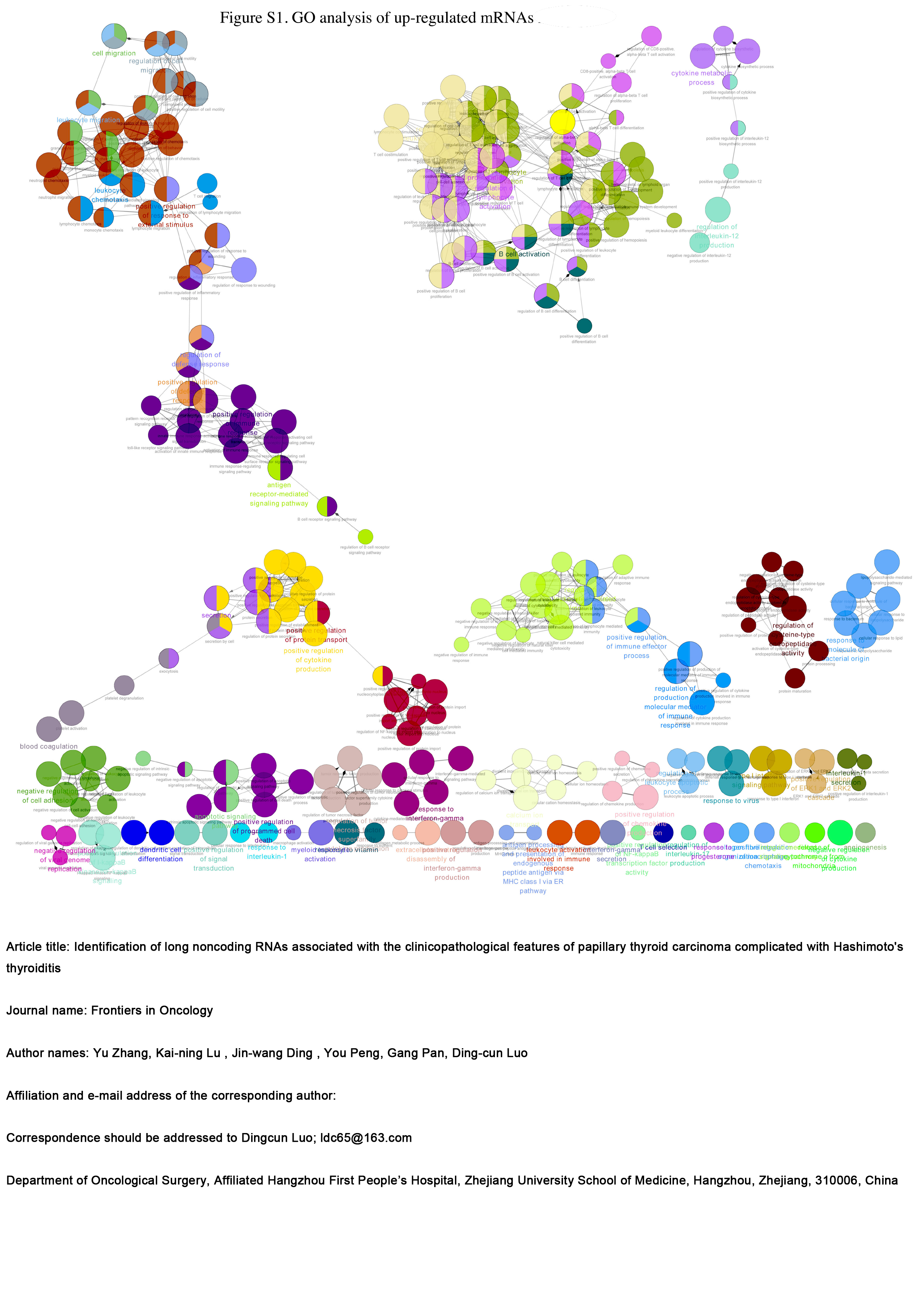

Supplement: Supplementary file 1 [file DataSheet_1.zip › revised-Supplementary Materials/revised-Supplementary Figure S1.tif]
